# Rare but elevated incidence of hematological malignancy after clozapine use in schizophrenia: a population cohort study

**DOI:** 10.1192/j.eurpsy.2025.2085

**Published:** 2025-08-26

**Authors:** F. T. T. Lai, Y. Hu, Y. Chai, L. Gao

**Affiliations:** 1 Department of Pharmacology and Pharmacy, University of Hong Kong; 2 Laboratory of Data Discovery for Health; 3 Department of Family Medicine and Primary Care, University of Hong Kong, Hong Kong, Hong Kong; 4 School of Public Health, Shenzhen University, Shenzhen; 5 School of Pharmacy, Xi’an Jiaotong University, Xi’an, China

## Abstract

**Introduction:**

Clozapine is widely regarded as a safe and highly efficacious psychotropic drug that is largely underused worldwide. Recent disproportionality analyses and nationwide case-control studies suggested a potential association between clozapine use and hematological malignancy (HM). Nevertheless, the absolute rate difference is not well-established due to the absence of analytic cohort studies. The clinical significance of such a potential risk remains unclear.

**Objectives:**

We aim to estimate the rate ratio and rate difference of HM associated with clozapine use.

**Methods:**

We extracted data from a territory-wide public healthcare database from January 2001 to August 2022 in Hong Kong to conduct a retrospective cohort study of anonymized patients aged 18+ with a diagnosis of schizophrenia who used clozapine or olanzapine (drug comparator with highly similar chemical structure and pharmacological mechanisms) for 90^+^ days, with at least two prior other antipsychotic use records within both groups. Weighted by inverse probability of treatment based on propensity scores, Poisson regression was used to estimate the incidence rate ratio (IRR) of HM between clozapine and olanzapine users. The absolute rate difference was also estimated.

**Results:**

In total, 9,965 patients were included, with 834 clozapine users. Both groups were followed up for an average of more than seven years. Clozapine users had a significant IRR of 2.22 (95% CI [1.52, 3.34]; p<0.001) for HM compared to olanzapine users. Absolute rate difference was estimated to be 57.40 (95% CI [33.24, 81.55]) per 100,000 person-years. Findings were consistent across sub-groups by age and sex in terms of effect size, although the IRR was non-significant for those aged 65 or older. Sensitivity analyses all supported the robustness of the results and showed good specificity to HM but no other cancers.

**Conclusions:**

Absolute rate difference in HM incidence associated with clozapine is small despite a twofold elevated rate. Given the rarity of HM and existing blood monitoring requirements, more restrictive indication for clozapine or special warnings may not be necessary.

**Disclosure of Interest:**

None Declared

